# Serological Evidence of Chikungunya Virus among Acute Febrile Patients in Southern Mozambique

**DOI:** 10.1371/journal.pntd.0004146

**Published:** 2015-10-16

**Authors:** Eduardo Samo Gudo, Gabriela Pinto, Sirkka Vene, Arcildo Mandlaze, Argentina Felisbela Muianga, Julie Cliff, Kerstin Falk

**Affiliations:** 1 National Institute of Health, Ministry of Health, Maputo, Mozambique; 2 The Public Health Agency of Sweden, Solna, Stockholm, Sweden; 3 Eduardo Mondlane University, Faculty of Medicine, Maputo, Mozambique; 4 Karolinska Institutet, Solna, Stockholm, Sweden; Florida Department of Health, UNITED STATES

## Abstract

**Background:**

In the last two decades, chikungunya virus (CHIKV) has rapidly expanded to several geographical areas, causing frequent outbreaks in sub-Saharan Africa, South East Asia, South America, and Europe. Therefore, the disease remains heavily neglected in Mozambique, and no recent study has been conducted.

**Methods:**

Between January and September 2013, acute febrile patients with no other evident cause of fever and attending a health center in a suburban area of Maputo city, Mozambique, were consecutively invited to participate. Paired acute and convalescent serum samples were requested from each participant. Convalescent samples were initially screened for anti-CHIKV IgG using a commercial indirect immunofluorescence test, and if positive, the corresponding acute sample was screened using the same test.

**Results:**

Four hundred patients were enrolled. The median age of study participants was 26 years (IQR: 21–33 years) and 57.5% (224/391) were female. Paired blood samples were obtained from 209 patients, of which 26.4% (55/208) were presented anti-CHIKV IgG antibodies in the convalescent sample. Seroconversion or a four-fold titer rise was confirmed in 9 (4.3%) patients.

**Conclusion:**

The results of this study strongly suggest that CHIKV is circulating in southern Mozambique. We recommend that CHIKV should be considered in the differential diagnosis of acute febrile illness in Mozambique and that systematic surveillance for CHIKV should be implemented.

## Author Summary

Chikungunya virus (CHIKV) is an emerging arbovirus that remains heavily neglected in Mozambique, and no recent study has been conducted. Between January and September 2013, four hundred acute febrile patients with no other evident cause of fever and attending a health center in a suburban area of Maputo city, Mozambique, were consecutively invited to participate. Paired acute and convalescent serum samples were drawn from each participant. Convalescent samples were initially screened for anti-CHIKV IgG, and if positive the corresponding acute sample was screened using the same test. Of the 209 patients from which paired samples was obtained, 26.4% (55/208) presented anti-CHIKV IgG antibodies in the convalescent sample. Seroconversion or a four-fold titer rise was confirmed in 9 (4.3%) patients. Overall our findings demonstrate that CHIKV is circulating in southern Mozambique and suggest that CHIKV should be considered in the differential diagnosis of acute febrile illness.

## Introduction

Chikungunya virus (CHIKV) is an arthropod borne virus (arbovirus) transmitted by *Aedes* mosquitoes and belonging to the *Togaviridae* family and *alphavirus* genus. Clinical presentation of CHIKV disease ranges from a self-limiting and undifferentiated febrile illness accompanied by exanthema, myalgia and headache to severe and debilitating polyarthritis and encephalitis. In a few cases death may occur [1–3].

CHIKV was described for the first time in 1952 during an outbreak in small villages in southern Tanzania close to the border with Mozambique. Cases were also reported in a few towns close to the border between Mozambique and Tanzania [2,4,5].

In recent years, the global health importance of CHIKV has increased significantly since the virus is a leading emerging vector borne infections worldwide [6,7]. Recently, several outbreaks have been reported in sub-Saharan Africa and South East Asia, including in Europe [1,2,7–10]. Outbreaks of chikungunya in temperate countries such as Italy represent a paradigm shift of mosquito-transmitted diseases [6,11]. The recent emergence of this virus in South America [12–15], made CHIKV probably the second most widespread arbovirus following dengue viruses. The main reasons for the resurgence of CHIKV worldwide include global warming, intense commercial trading, deforestation, and changes in the ecology and geographical distribution of *Aedes* mosquitoes [6,7].

Although the initial discovery of CHIKV is geographically linked to Mozambique[5], the disease has been heavily neglected locally for the followings reasons: i) non-specific clinical presentation, ii) lack of local diagnostic capacity for CHIKV confirmation and iii) lack of epidemiological data on the chikungunya burden. The scarce available information of CHIKV in Mozambique is more than 40 years old [16]. Due to the lack of recent serological and epidemiological data, Mozambique has been repeatedly excluded from the list of affected countries in reports describing the global distribution of CHIKV [1,17,18], and consequently, the country is frequently considered free of this virus. Evidence of CHIKV in the neighboring countries is also very scarce, and most of available data are old [19–22].

Mozambique is located on the south-eastern coast of Africa, with more than 2,500 Km of coast and represents a strategic hub for the region. The concern that CHIKV would currently constitute an important cause of acute febrile illness in Mozambique has recently increased since recent entomological research conducted and published by our group demonstrated an abundance of *Aedes aegypti*, the vector for CHIKV in several geographical areas in Mozambique, including Maputo city in southern Mozambique where no previous reports of CHIKV exist [23]. Furthermore, *Aedes* was involved in the 2014 outbreak of dengue in two provinces in northern Mozambique [24] and a recent case of concomitant *Plasmodium falciparum* and CHIKV infections in an adult patient living in northern Mozambique was laboratory confirmed in a private clinic in South Africa [25]. In addition, the largest CHIKV outbreak reported in history occurred during 2005–2007 in several islands situated in the Indian Ocean and Mozambique Channel, geographically close to Mozambique [26,27] and recently, several cases of CHIKV have been reported in Tanzania[28,29] and Angola[30], respectively.

CHIKV may also have an impact on efforts to control malaria and other vector borne acute febrile illnesses transmitted by *Aedes*, such as dengue, in countries endemic for these diseases, including Mozambique, as the clinical presentation of CHIKV overlaps those of other febrile conditions. In these settings, CHIKV may circulate unsuspected, leading to over-diagnosis of malaria and overuse of antimalarial drugs[31].

Although the available evidence strongly suggests that CHIKV circulates in Mozambique, the information on the epidemiology of the disease is dated as well as insufficient, and no study has as yet been conducted in southern Mozambique. This study was therefore conducted with the aim to provide recent information on the frequency of CHIKV in acute febrile patients in southern Mozambique.

## Materials and Methods

### Study design and study setting

**Fig 1:**
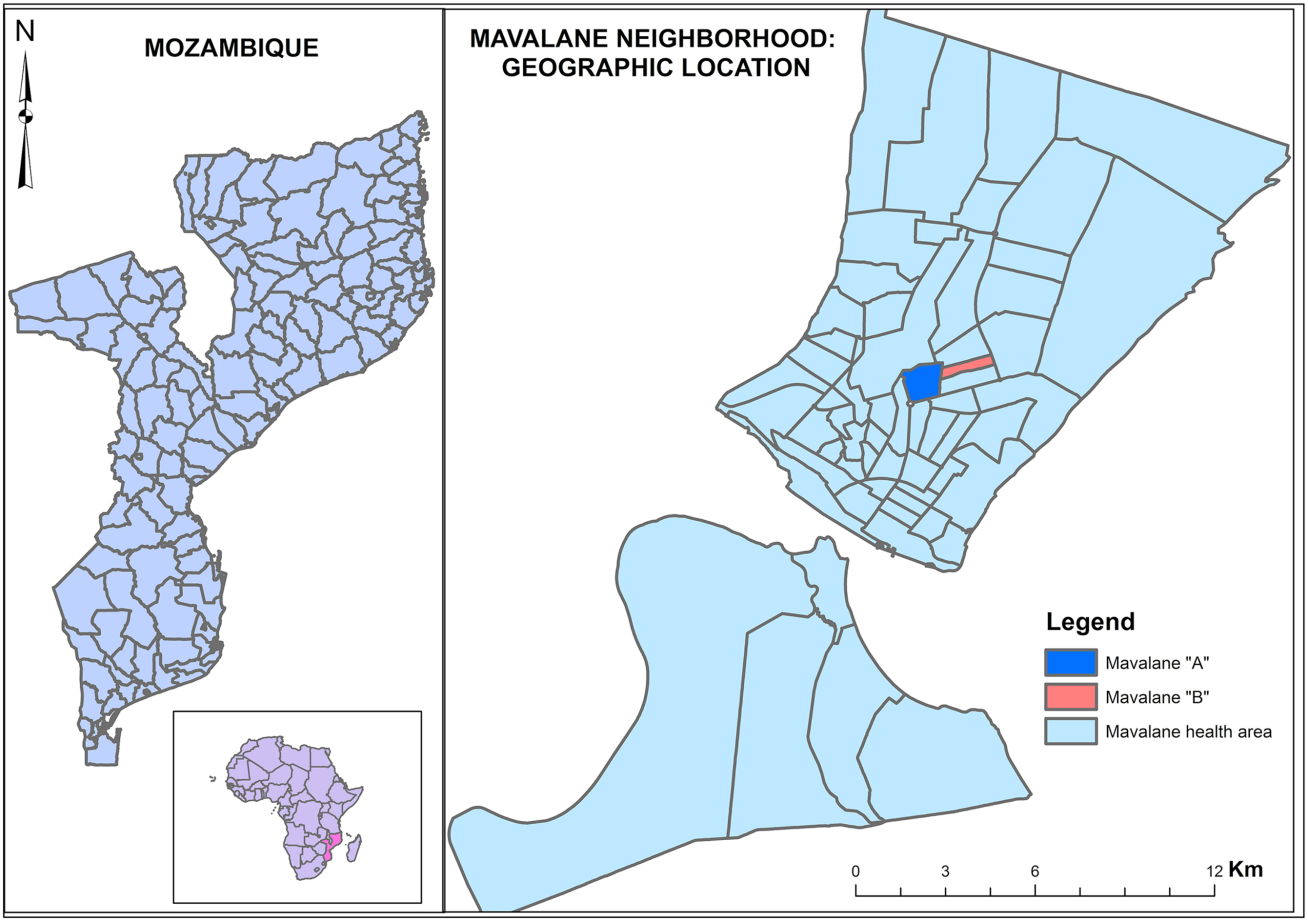
Geographical representation of study area. Left panel shows the geographical localization of Mozambique in south east Africa (Mozambique is highlighted in pink) and map of Mozambique. Right panel is the representation of the neighborhood of Mavalane health area covered by the Mavalane health center. Mavalane health area comprises two geographical and well delimited areas, namely, Mavalane “A” (dark blue) and Mavalane “B” (pink) neighborhood.

This study was conducted at the Mavalane Health Center, a primary health care facility located in a large suburban area in Maputo City in southern Mozambique (see Fig 1). The study area is characterized by poverty, poor sanitation, and an extremely high population density. The prevalence of malaria is high in this setting. The main sources of income are from the informal sector and small business. The rainy season extends from November through March.

Eligibility criteria included presence of acute febrile syndrome (axillary temperature > 37.5°C), age > 5 years and ability to provide a written informed consent. Exclusion criteria. Pregnant women, individuals with psychiatric disease, individuals with a readily identifiable focus of infection, such as otitis media, sinusitis, purulent pharyngitis, cellulites, urinary tract infection, dental abscess, septic arthritis, pneumonia or pelvic inflammatory disease were excluded from participation in this study

All eligible patients attending the Mavalane Health Center between January and September 2013 were consecutively invited to participate and enrolled. At the enrollment visit, a blood sample (acute sample) was requested from each patient and all of them were requested to return to the health facility after three weeks for collection of a convalescent sample. A few days before the scheduled date for the convalescent visit, the investigator made phone call to remind each participant of their appointment. Patients with malaria positive test results were not excluded.

### Case definitions

The WHO guidelines [32] for a laboratory confirmed CHIKV infection include the isolation of virus as well as the demonstration of virus specific IgM antibodies or a four-fold titer rise of IgG in samples collected at least three weeks apart.

Patients with anti-CHIKV IgG in both acute and convalescent samples, but lacking a four-fold increase in titer were classified as having previous exposure to CHIKV.

The absence of anti-CHIKV IgG was defined as no CHIKV infection.

### Ethics statement

This study was approved by the National Bioethical Committee in Mozambique (Ref: IRB00002657) prior to initiation. Written and informed consent was requested from each patient before enrollment.

### Questionnaire

From each participant who gave the written consent to participate, clinical, demographic and epidemiological data was collected using a questionnaire. The collected information included age, gender, residence and clinical presentation.

### Blood collection

Venous blood was collected from all acute febrile patients who consented to participate in the study. Whole blood was collected aseptically; 5ml into a K_3_EDTA tube and 5ml into a Serum Separation Tube (both from BD Vaccutainer, USA). Specimens were delivered to the laboratory within four hours of collection for centrifugation, separation and storage at -70°C.

### Laboratory testing

#### Blood smear microscopy

Upon delivery of samples to the laboratory, a thick blood smear was mounted from anticoagulated whole blood. All blood smears were stained using the Giemsa protocol and screened for *Plasmodium falciparum*, *P*. *malarie*, *P*. *ovale* and *P*. *vivax* using light microscopy. Parasite density was estimated by means of a semi-quantitative scale.

#### Chikungunya virus immunofluorescence

**Fig 2:**
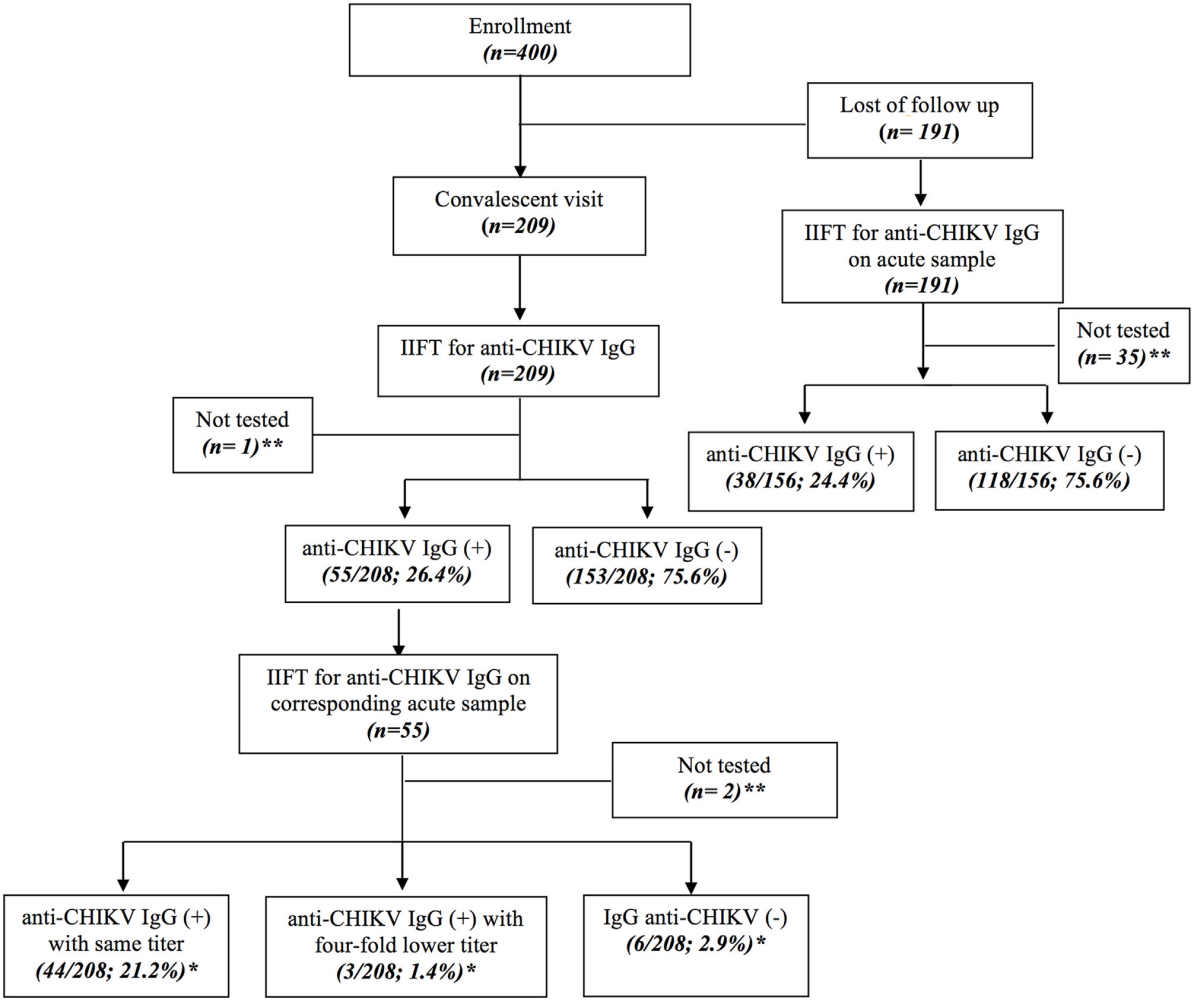
Flowchart of recruitment of study participants and sample testing. Study participants were split into two groups, those who returned to the convalescent visit (left side of the flowchart) and those who were lost at follow-up (right side of the flow chart). The left side of the flow chart demonstrates that out of 400 recruited patients, 209 returned for the follow-up visit and their convalescent samples were initially screened for anti-CHIKV IgG. Out of 208 tested samples, 55 (26.4%) were positive and 153 (75.6%) were negative. Further, the corresponding 55 acute samples of the positive samples were screened for anti-CHIKV IgG. A total of 44 (21.2%) were positive with stationary titers, 3 (1.4%) were positive with four fold titer rise and 6 (2.9%) seroconverted. The right side of the flow chart demonstrates that 191 individuals did not return for their follow-up; 156 acute samples were available for testing and of these 38 (24.4%) and 118 (75.6%) were positive and negative, respectively. * Percentage against the total number of patients who returned to convalescent visit. ** Samples not tested because of insufficient volume

Convalescent serum samples were initially screened for anti-CHIKV IgG at a 1:20 dilution using a commercial indirect immunofluorescence test (IIFT) (EUROIMMUN AG, Lübeck, Germany). If the screening result of convalescent sample was positive, corresponding acute and convalescent samples were tested in parallel in a dilution series to determine possible sero-conversions or titer rises. Acute samples from patients who did not return for a convalescent visit were also screened for anti-CHIKV IgG (Fig 2). The manufacturer claims that sensitivity and specificity of the assay is 95% and 100%, respectively.

Acute samples of patients with CHIKV infection were also tested for IgM anti-CHIKV, using a commercial indirect immunofluorescence test (IIFT) (EUROIMMUN AG, Lübeck, Germany).

As per WHO guidelines[32], acute CHIKV infection was defined using the followings criteria: i) presence of CHIKV anti-IgM or ii) seroconversion of CHIKV anti-IgG from acute to convalescent sample or ii) at least a four-fold titer rise of CHIKV anti-IgG antibodies between acute and convalescent sample.

#### Virus isolation

Attempts to isolate virus from acute serum samples were made in six cases where a sero-conversion to CHIKV was observed. Samples were diluted 1:10 in tissue culture medium and 100 μl was inoculated on Vero cells in 2cm^2^ tissue culture tubes. Following incubation for 1 hour at +37°C, the inoculum was removed, 2 ml of tissue culture medium added, and tubes were incubated at +37°C for 8 days, and inspected for cytopathogenic effect daily.

### Data analysis

Sample size to estimate seroprevalence of Chikungunya (IgG) in febrile patients was calculated based on the following assumptions, i) expected seroprevalence (frequency of IgG anti-CHIKV) of 15% using as the basis for comparison, the seroprevalence reported in recent studies conducted in Tanzania ii) precision rate of 3.5%. With these assumptions, the final size of the sample was 400 febrile patients. Data was analyzed using the statistics package STATA 9.0 (College Station, Texas: StataCorp, USA, 2005). The Kruskal Wallis test was used to compare the study groups regarding numerical variables. Associations between categorical variables were determined using the Pearson Chi-square test. Logistic regression analysis was employed to determine the variables independently associated with CHIKV antibodies, controlling for confounders. The analysis was built using the backwards stepwise method and Log Likelihood Ratio Test. For this purpose, all variables with a P-value less than 0.1 on univariate analysis were included in the initial multivariate model. A p value < 0.05 was considered of statistical significance in the final model.

## Results

### General characteristics of study participants

Four hundred patients were enrolled between January and August 2013. Of these, 209 (52.3%) returned for the follow-up visit, although efforts were undertaken by the investigators to recall each participant by phone call to return for their convalescent follow up (see Fig 2). The reason why 47.7% of patients did not return for their convalescent visits are unknown, but information provided by clinicians mention that after recovery patients rarely return to the health facility for convalescent follow up.

The median age of study participants was 26 years (IQR: 21–33 years) and 57.5% (224/391) were female.

The average time between onset of symptoms and collection of the acute sample was 24h. In regard to convalescent sample, the average number of days from disease onset was 25 days.

### Laboratory results

Convalescent samples from the 209 patients who returned for the follow-up visit were tested for anti-CHIKV IgG using IIFA, 55 (26%) of which were positive, 153 (74%) tested negative and one sample was not tested due to insufficient volume.

Subsequently, the corresponding acute samples (n = 55) from the patients who tested positive in their convalescent sample were tested for anti-CHIKV IgG. Seroconversion or a four-fold titer rise representing the definition criteria for acute CHIKV infection, occurred in nine (4.3%) patients. Anti-CHIKV IgM testing was performed in these 9 serum samples and all were negative. Virus isolation attempts from six CHIKV IgG negative samples were unsuccessful.

Fig 2 demonstrates that anti-CHIKV IgG measurement was also performed in acute samples from patients who did not return for their follow-up visit and the positivity rate was similar to that observed for convalescent samples (24.4%, 38/156 for acute sample of patients who did not return for the follow-up visit *versus* 26.4%, 55/208 for convalescent samples from patients who returned for the follow- up visit).

Results of malaria blood smears were available for 328 patients, of which 26 were smear positive, yielding an overall positivity rate for malaria of 7.9%.

### Clinical and demographic characteristics of study groups

Based on IIFA for anti-CHIKV IgG, patients who returned for the follow-up visit were stratified into three main groups, i.e., i) acute infection (9/209, 4.3%), ii) previous exposure (44/209, 21.2%) and iii) negative CHIKV infection (153/209, 73.2%).

Febrile patients with acute infection, previous exposure and negative CHIKV infection were similar in terms of age, gender and clinical presentation as shown in Table 1. A few participants had a history of recent international travel, and the destination was South Africa for all of them. Arthralgia was de most common symptom in the patients with CHIKV infection, but in the other two groups, headache was the more common symptom. No patients presented any form of hemorrhage.

**Table 1:** Clinical and demographic characteristics of study participants stratified by CHIKV infection status.

Characteristics	Acute Infection^1^ n (%)	Previous exposure^2^ n (%)	Negative CHIKV Infection^3^ n (%)	*p-value*
**Total**	n = 9	n = 44	n = 153	
**Age**				
Median	30	28	27	0.978
IQR	17–47	22–31	21–35	
**Gender**				
Male	3 (33.3)	16 (38.6)	70 (46.4)	0.560
Female	6 (66.7)	27 (61.4)	81 (53.6)	
**Recent international travel**	0 (0.0)	1 (2.3)	3 (2.0)	0.968
**Chills**	4 (44.4)	22 (53.7)	90 (58.8)	0.615
**Pruritus**	2 (22.2)	3 (6.8)	14 (9.2)	0.346
**Headache**	4 (44.4)	34 (77.3)	103 (67.3)	0.215
**Arthralgia**	5 (55.6)	17 (38.6)	46 (30.1)	0.192
**Skin Rash**	2 (22.2)	4 (9.1)	13 (8.5)	0.384
**Myalgia**	4 (44.4)	17 (38.6)	55 (36.0)	0.845
**Abdominal pain**	4 (44.4)	15 (34.1)	64 (41.8)	0.632
**Vomiting**	3 (37.5)	4 (9.1)	25 (16.5)	0.111
**Diarrhea**	2 (22.2)	4 (9.1)	15 (10.1)	0.485
**Hemorrhage**	0	0	0	-

^**1**^ **Acute infection:** Seroconversion of IgG anti-CHIKV or four fold increase of IgG anti-CHIKV titter between acute and convalescent sample

^**2**^ **Previous exposure:** Presence of IgG anti-CHIKV in the acute and in the convalescent sample < 4 fold increase in the titter between the samples

^**3**^ **Negative CHIKV Infection:** IgG anti-CHIKV negative

## Discussion

In Mozambique and other malaria endemic countries, the lack of epidemiologic information concerning the etiology of acute febrile illness results in over-diagnosis and over-treatment of malaria and increased mortality related to wrong therapeutic intervention[31]. Although available evidence from other sub-Saharan countries close to Mozambique[1], strongly suggests that CHIKV would be among the main causes of acute febrile illness, this virus is heavily neglected in Mozambique and no data has been published reporting CHIKV in Mozambique over the last 40 years. This study was therefore conducted with the aim to determine the burden of CHIKV in acute febrile patients in Mozambique and represents the first sero-epidemiological investigation of CHIKV in southern Mozambique. Methodologically, this study represents an improvement over the few available studies conducted more than 40 years ago[33], since, for the first time, paired acute and convalescent samples were collected from each participant and tested for CHIKV antibodies.

Patients with acute febrile illness were invited to participate in this study and their samples were serologically screened for CHIKV IgG antibodies. We used the WHO criteria to define recent infection based on measurement of CHIKV IgG antibodies in acute and convalescent sample, as previously mentioned. CHIKV IgG seroconversion and titer rises were observed in 4.3% (9/208) of patients. This finding is similar to that of a recent study conducted in Tanzania, which found a frequency of acute CHIKV infection of 4.3% among febrile patients, but is slightly lower of than reported in another study conducted in northern Tanzania, which found a frequency of acute infection of 7.9%[34]. Altogether, these findings reinforce our previous hypothesis that CHIKV is prevalent in Mozambique[28]. Our result is not surprising since a recent paper published by our group demonstrated that *Aedes* mosquitoes is abundant in Maputo city. In our study, we demonstrated that *Aedes* was present in 66.4% (83/125) of mosquito reservoirs that we observed[23]. Furthermore, CHIKV has been involved in outbreaks in Southern Africa [1,28] and the largest outbreak reported in history occurred in several islands situated in the Indian Ocean and Mozambique Channel, close to Mozambique [26,27]. In addition, a recent case of co-infection by malaria and CHIKV in an adult patient living in Pemba, situated in the northern Mozambique, was recently confirmed at a private laboratory in South Africa[25].

We found a frequency of previous exposure to CHIKV in 24.6% (55/208) of patients who returned for a convalescent visit (see Fig 2). This demonstrates that exposure to CHIKV is common and strongly suggests that CHIKV would be an important cause of acute fever, causing unsuspected sporadic cases or outbreaks and consequently may have been overlooked and mis-diagnosed as malaria. We hypothesized that this not only hampers the national efforts to control malaria, but also contributes to the increased risk of mortality related to the administration of incorrect treatment.

Although this study was conducted in a small geographical area in southern Mozambique, we can speculate that CHIKV is also circulating in other geographical areas of the country, since a recent entomologic investigation demonstrated that the density of *Aedes* mosquitos in two provinces situated in northern Mozambique was much higher than in Maputo[23] and that previous reports demonstrated that CHIKV caused outbreaks or sporadic cases in northern Mozambique[5,33]. Moreover, recent papers reported circulation of CHIKV in three different regions in Tanzania, a neighboring country in the northern Mozambique[28,29,34].

Data from our study show that the clinical presentation of patients with acute CHIKV infection was similar of that from patients with previous exposure or with no CHIKV infection. These findings are similar to those from previous studies in the Sub-Saharan region of Africa[27].

In order to understand the burden of malaria *versus* CHIKV, patients suspected of malaria were not excluded from this study and our results demonstrated that *P*. *falciparum* was confirmed in 7.9% (26/302) of febrile patients. This highlights the concern that in several settings in Mozambique and other sub-Saharan countries, malaria is no longer the main cause of febrile disease, in a context where the burden of other emerging diseases is increasing [35]. The serious lack of epidemiological data regarding the etiology of acute fever in Mozambique and other sub-Saharan countries represents a serious challenge for the proper management of these patients and is considered the main cause of over-diagnosis and over-treatment of malaria[31]. Further studies should be conducted in order to better understand the epidemiology of acute fever and improve the algorithms for management of acute febrile illness, not only in Mozambique, but in other sub-Saharan countries.

Acute samples from patients with acute CHIKV infection were negative for anti-IgM CHIKV. This was not a surprising finding as acute samples were in average collected 1 day after onset of symptoms, and thus likely before sero-conversion of IgM. Virus isolation assay was performed on six acute samples from patients with CHIKVIgG seroconversions, but all were negative. This was also not surprising, as serum samples were poorly managed during transportation and storage, which may have resulted in viral RNA degradation.

We acknowledge the limitations of our study, such as the rate of loss of participants for the follow up visit. Unfortunately, it is well known that acute febrile patients rarely return for convalescent visits, since most febrile diseases are of short duration, and after recovery the patients return to their routine activities with no interest in follow up visits. Serological cross-reactivity between arboviruses has been considered a limitation in several serological assays. In this regard, ten samples reactive for IgG anti-CHIKV were also tested for Sindbis virus IgG antibodies using an in-house ELISA, and all were non-reactive. In addition, the manufacturer claims that the specificity of EUROIMMUNE immunofluorescence assay is very high (100%).

In conclusion, our data demonstrate that CHIKV infections are more frequent than thought, and thus we recommend that: i) chikungunya should be considered as a differential diagnosis of acute febrile illness to reduce malaria over-diagnosis and over- treatment with antimalarial drugs, ii) sentinel surveillance systems for CHIKV should be implemented and expanded to other regions in the country and iii) further entomological studies should be conducted to better understand the distribution of the CHIKV vector in Mozambique.

## Supporting Information

S1 Checklist STROBE checklist. (DOC)
